# MmuPV1 E7 promotes phenotypes associated with “high-risk” HPV infection in mouse keratinocytes

**DOI:** 10.1128/jvi.01097-25

**Published:** 2025-10-30

**Authors:** Kayla R. Duxbury, Liyan Zhang, Laura K. Muelhbauer, Mitchell Hayes, Joshua J. Coon, Megha Padi, James C. Romero-Masters

**Affiliations:** 1 Department of Biomedical Sciences and Pathobiology, Virginia-Maryland College of Veterinary Medicine, Virginia Tech, Blacksburg, Virginia, USA; 2 Center for One Health Research, Virginia Tech 1757, Blacksburg, Virginia, USA; 3 Center for Emerging, Zoonotic, and Arthropod Borne Diseases, Virginia Tech, Blacksburg, Virginia, USA; 4 Department of Chemistry and Biomolecular Biology, University of Wisconsin-Madison 5228, Madison, Wisconsin, USA; 5 McArdle Laboratories for Cancer Research, University of Wisconsin School of Medicine and Public Health, Madison, Wisconsin, USA; 6 Morgridge Research Institute 145254, Madison, Wisconsin, USA; 7 Department of Molecular and Cellular Biology, University of Arizona 8041, Tucson, Arizona, USA; College of Agriculture & Life Sciences, University of Arizona, Tucson, Arizona, USA

**Keywords:** keratinocyte biology, E7, MmuPV1, HPV

## Abstract

The E7 oncoprotein of mouse papillomavirus (MmuPV1) plays a pivotal role in both viral infection and cancer development. While earlier studies have identified key cellular targets of MmuPV1 E7, such as pRB and PTPN14, the broader impact of MmuPV1 E7 on keratinocyte homeostasis and shared activities with human papillomavirus (HPV) E7 remains unclear. In this study, we employed proteomic and transcriptomic analyses using our established mouse keratinocyte model—previously instrumental in uncovering a novel function of MmuPV1 E6—to investigate the biological consequences of MmuPV1 E7 expression in mouse keratinocytes. Our findings reveal that MmuPV1 E7 induces cellular changes reminiscent of those driven by “high-risk” HPV infection implicated in cervical cancer. Notably, MmuPV1 E7 did not activate canonical E2F-responsive gene expression or promote proliferation, reinforcing the idea that MmuPV1 E6 is the primary driver of cell cycle activation. However, MmuPV1 E7 expression led to a significant accumulation of stress keratin 17, a marker associated with immune evasion and elevated in both HPV16 transgenic models and MmuPV1 infections. Additionally, we observed enhanced PI3K-AKT-mTOR signaling, with increased levels of phosphorylated S6 kinase and heightened sensitivity to epidermal growth factor stimulation. Collectively, these results underscore the role of MmuPV1 E7 in promoting oncogenic phenotypes and highlight its relevance as a model for studying the molecular underpinnings of “high-risk” HPV-driven disease.

**IMPORTANCE:**

In this study, we determined the ability of the MmuPV1 E7 oncoprotein in promoting disruption of keratinocyte homeostasis in mouse keratinocytes. Using a multiomics approach, we observed that MmuPV1 E7 promoted several phenotypes associated with “high-risk” human papillomavirus (HPV) infection. Specifically, we confirmed that MmuPV1 E7 does not increase E2F-responsive gene expression and proliferation of mouse keratinocytes. We did find that MmuPV1 E7 was able to increase the expression of stress keratin 17, which promotes immune evasion in papillomavirus infections. Finally, MmuPV1 E7 showed increased expression of genes associated with PI3K-AKT-mTOR signaling. Consistent with this observation, MmuPV1 E7-expressing mouse keratinocytes had elevated phosphorylation of S6 kinase. We also found that MmuPV1 E7 potentiates this signaling through increased sensitivity to epidermal growth factor stimulation. Our collective data show that MmuPV1 E7 promotes several phenotypes associated with “high-risk” HPV infection and cancers.

## INTRODUCTION

Human papillomaviruses (HPVs) are a large family of non-enveloped double-stranded DNA viruses with genomes ~8 kb in size (1, 2). HPVs infect stratified squamous epithelium and are divided into two different groups, mucosal and cutaneous, based upon the type of stratified squamous epithelium they infect (1, 2). A subset of mucosal HPVs (“high-risk” HPVs) are causative agents of cervical and head and neck cancers and cause 5% of the world’s total cancer burden (3, 4). In contrast, “low-risk” mucosal and cutaneous HPVs typically cause benign neoplastic disease, i.e., genital and skin warts, in patients (5–7). A small subset of cutaneous HPVs is associated with non-melanoma skin cancer in long-term immunosuppressed transplant patients and patients with the genetic disorder epidermodysplasia verruciformis (8–10).

Many decades of work have solidified the role of HPV in cancer development through activities of the virally encoded E6 and E7 oncogenes, in particular “high-risk” HPVs. The ability of “high-risk” HPV E6 and E7 to immortalize cells has been linked to inhibition of tumor suppressor signaling in cells (11–13). “High-risk” HPV E6’s most well-characterized activities are promoting degradation of p53 and increased telomerase reverse transcriptase (TERT) activity, which leads to prolonged survival of HPV-infected cells (14–19). “High-risk” HPV E7’s most well-characterized activities include the degradation of the retinoblastoma tumor suppressor (pRB) and the cellular protein tyrosine phosphatase non-receptor 14 (PTPN14) (20–24). The inactivation of pRB by HPV E7 promotes constitutive activation of E2F transcription factors and leads to dysregulation of cellular proliferation (25). The ability of HPV E7 to promote degradation of PTPN14 has been linked to resistance to anoikis and activation of oncogenic Yes-associated protein signaling (22–24). However, the significance of “high-risk” HPV E7’s activities that contribute to disease development *in vivo,* including cancer, has been limited to the transgenic mouse model system (26–33). In this model system, the most well-characterized activity of “high-risk” HPV E7 has been E7’s interaction with pRB family members (33, 34). In contrast, the role of PTPN14 in the transgenic animal model setting has never been studied. Cutaneous HPV E7 has been shown to interact with tumor suppressors pRB and PTPN14 (35–39). In contrast to “high-risk” HPV E7, cutaneous HPV E7 degradation of pRB is observed but not uniform among the various subtypes (36, 37). Cutaneous HPV transgenic models have been used to study the role of cutaneous HPV E6 and E7 *in vivo* (40–42). However, cutaneous HPV E6 and E7 in general are understudied compared to their “high-risk” HPV counterparts.

The species-specific nature of papillomaviruses has inhibited the ability to study the contributions of HPV E6 and E7 to an infection *in vivo*. The murine papillomavirus, MmuPV1, discovered in 2011, enables us to study a papillomavirus infection within a genetically tractable preclinical animal model (43). MmuPV1 infects laboratory mice (*Mus musculus*), at all the same anatomical sites as HPV, and causes lesions that can progress to cancer, including skin, cervicovaginal tract, anal tract, and oral cavity (44–51). These observations make MmuPV1 an attractive model for studying HPV-associated pathogenesis. MmuPV1 is a member of the *Pipapillomavirus* genus and is more genetically similar to cutaneous HPV genotypes. The MmuPV1 E6 and E7 oncoproteins are capable of binding to known cellular binding partners of HPV E6 and E7 (52–54). However, the biochemistry of these interactions more closely mimics the cutaneous HPV E6 and E7 oncoproteins (52–58). MmuPV1 E7 does interact with the tumor suppressor pRB and PTPN14 (53, 54). MmuPV1 E7’s interaction with pRB is not facilitated through an LXCXE motif but instead through amino acids in the C-terminus of MmuPV1 E7, which has been observed in HPV E7s that lack LXCXE motifs and canine papillomavirus (53, 55). While the interaction between MmuPV1 E7 and pRB is distinctly different from the “high-risk” HPVs, previous work has shown that the interaction between MmuPV1 E7 and pRB plays a key role in pathogenesis (33). However, the impact of MmuPV1 E7 on pRB biology has remained elusive. MmuPV1 E7 interacts with the tumor suppressor PTPN14 using similar amino acids as “high-risk” HPV E7, but it remains unclear if MmuPV1 E7 promotes degradation of PTPN14 like the “high-risk” HPV E7s (54). Previous work has shown that MmuPV1 E7, like HPV E7, promotes inhibition of keratinocyte differentiation through its interaction with PTPN14 (22, 23, 35, 54). While recent work has made significant progress in understanding the role of MmuPV1 E7 and the cellular interacting partners that play key roles in pathogenesis, our understanding of the impact of MmuPV1 E7 on keratinocyte biology remains limited to inhibition of differentiation.

In this study, we utilized a multiomics approach to identify the impact on cellular homeostasis in mouse keratinocytes (MKs) in the presence of MmuPV1 E7. Specifically, we performed quantitative mass spectrometry for proteomics analysis and RNA-seq for transcriptomic analysis. Both our RNA-seq and quantitative mass spectrometry analyses revealed that MmuPV1 E7 significantly alters cellular homeostasis and promotes phenotypes that have been associated with HPV infection and cancer, including “high-risk” HPV-associated disease. We observe a mild but significant increase in proliferation-associated genes. However, we do not see an increase in the proliferation rate of MmuPV1 E7-expressing MKs, nor did we see a significant increase in classic E2F-responsive gene expression. Interestingly, we observed a significant increase in the transcription of stress keratins, including keratin 17 (K17), which has been shown to reduce T cell recruitment to MmuPV1-infected warts (59). We observed a trend in increased protein steady-state levels of stress keratins 6a, 6b, and 16, whereas K17 showed a significant increase in the steady-state protein levels. Finally, we observed a significant increase in the activation of mTOR signaling in MmuPV1 E7-expressing MKs but not MmuPV1 E6-expressing MKs. We were able to determine that the increase in mTOR signaling is potentially due to increased sensitivity to epidermal growth factor receptor (EGFR) signaling as MmuPV1 E7-expressing MKs have an elevated response to EGF stimulation following EGF deprivation. Based upon these results, we conclude that MmuPV1 E7, like MmuPV1 E6, does significantly contribute to phenotypes associated with papillomavirus infection and pathogenesis, including “high-risk” HPV infection.

## RESULTS

### MmuPV1 E7 alters cellular homeostasis in mouse keratinocytes

**Fig 1:**
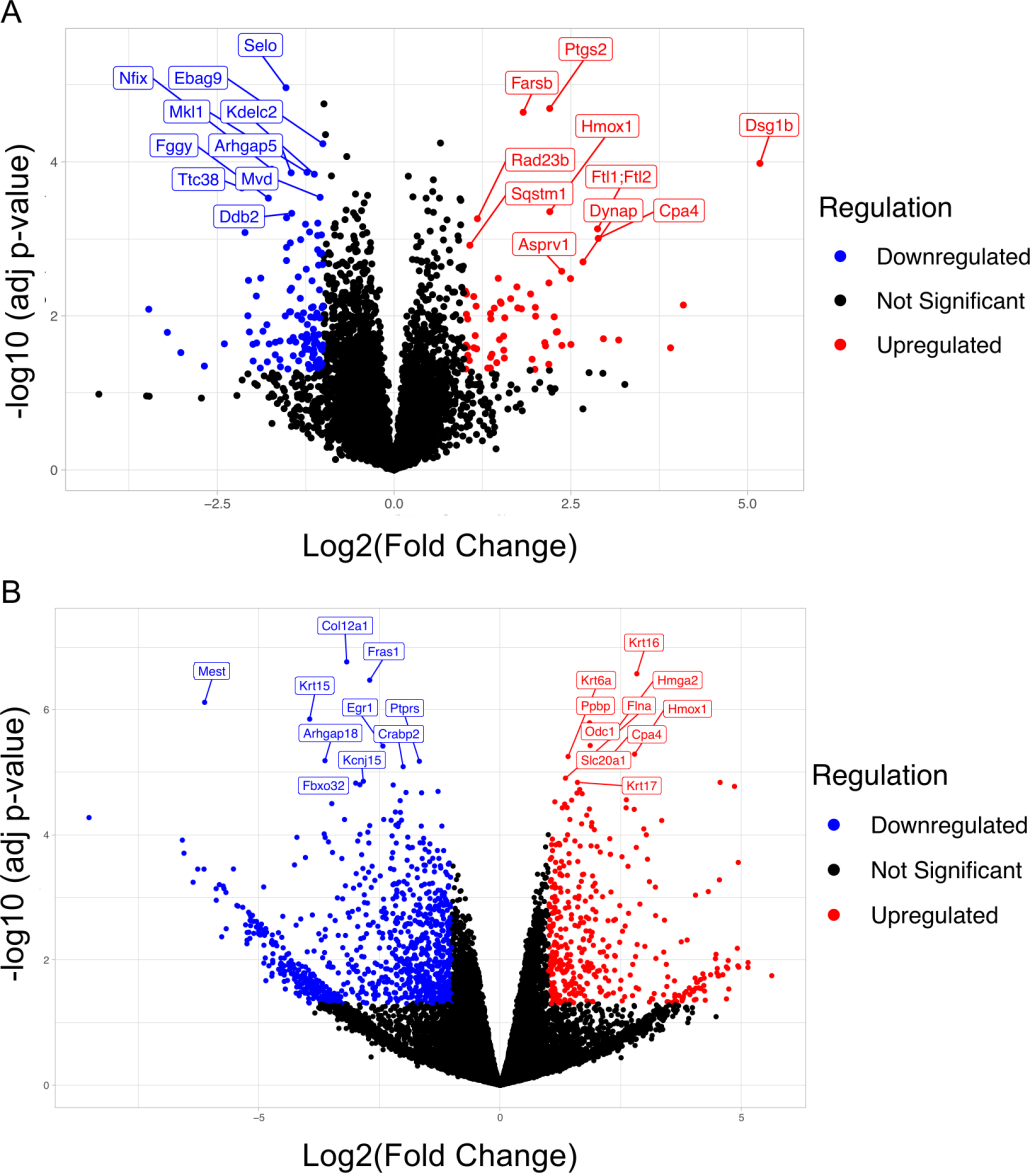
MmuPV1 E7 alters the cellular proteome and transcriptome. (**A**) Volcano plot of quantitative mass spectrometry analysis performed comparing MmuPV1 E7-expressing and vector control mouse keratinocytes. Red dots are proteins with a log2(FC) > 1 and adjusted *P*-value <0.05. Blue dots are proteins with log2(FC) < −1 and adjusted *P*-value <0.05. Black dots are proteins that do not meet the requirements stated above. The top 10 most upregulated proteins (red) and top 10 most downregulated proteins (blue) are shown. (**B**) Volcano plot of RNA-seq analysis performed comparing MmuPV1 E7-expressing and vector control mouse keratinocytes. Red dots are genes with a log2(FC) > 1 and adjusted *P*-value <0.05. Blue dots are genes with log2(FC) < −1 and adjusted *P*-value <0.05. Black dots are genes that do not meet the requirements stated above. The top 10 most upregulated transcripts (red) and top 10 most downregulated transcripts (blue) are shown.

Our previous work has found that MmuPV1 E7 interacts with known cellular targets of HPV E7, including pRB and PTPN14 (53, 54). Through these studies, we found that both the interactions with pRB and PTPN14 contribute to viral pathogenesis (53, 54). However, our understanding of the impact of MmuPV1 E7 on cellular homeostasis and pathogenesis is limited to MmuPV1 E7’s interaction with PTPN14 restricting differentiation. To address this gap in knowledge, we performed two different unbiased omics analyses, RNA-seq (transcriptomics) and quantitative mass spectrometry (proteomics), to gain a broad understanding of the impact of MmuPV1 E7 on cellular homeostasis. For this analysis, we generated mouse keratinocyte cell strains (MKs) from neonate mouse skin (days 1–4 postpartum). Following the establishment of MKs, we transduced early-passage MKs (passage <5) with a retroviral vector that encoded the MmuPV1 E7 gene (pLXSP mE7) or an empty control vector, and cells were selected for MmuPV1 E7 expression using puromycin. MmuPV1 E7 expression was validated using reverse transcription-PCR (RT-PCR), as no antibody for immunoblot analysis is available for MmuPV1 E7. Following confirmation of MmuPV1 E7 expression, we subjected early-passage MmuPV1 E7-expressing and vector control MKs (passages 2 and 3) to transcriptomic and proteomic analysis. Our analyses found that MmuPV1 E7 significantly altered cellular homeostasis, with greater than 700 genes significantly altered transcriptionally (Table S1) and greater than 170 proteins (Table S2) significantly altered at the steady-state level (Table 1). One gene, Cth, and its corresponding protein, cystathionine gamma-lyase, were found to be conflicting between the two analyses (Table S3). However, MmuPV1 E7’s impact on cellular homeostasis is less significant compared to MmuPV1 E6, both transcriptionally and on protein steady state (Table 1) (57). We generated a volcano plot to highlight the top 10 most significantly upregulated and top 10 most significantly downregulated proteins from our proteomics analysis (Fig. 1A). Several of these proteins are implicated in cervical cancer, including Ptgs2 (up), Hmox1 (up), Mkl1 (down), and Arhgap5 (down) (60–63). We performed a similar analysis on our RNA-seq analysis and identified several genes that are also implicated in cervical cancer and HPV infection, including Krt16 (up), Krt6a (up), Hmox1 (up), and Krt15 (down) (Fig. 1B) (62, 64, 65). These observations suggest that MmuPV1 E7 promotes cellular changes that are observed in HPV+ cancers, including “high-risk” HPV+ cancers.

**TABLE 1:** MmuPV1 E7 alters cellular proteome and transcriptome^*a*^

	RNA-seq		Proteomics	
	E7	E6	E7	E6
Upregulated	281	791	64	426
Downregulated	515	1,162	112	516

^*a*^ The numbers of differentially expressed genes and proteins in our RNA-seq and quantitative mass spectrometry, respectively, are shown. The comparisons performed include MmuPV1 E7 vs vector control MKs and MmuPV1 E6 versus vector control MKs (already published data) (57).

### MmuPV1 E7 promotes phenotypes associated with “high-risk” HPV infection

**Fig 2:**
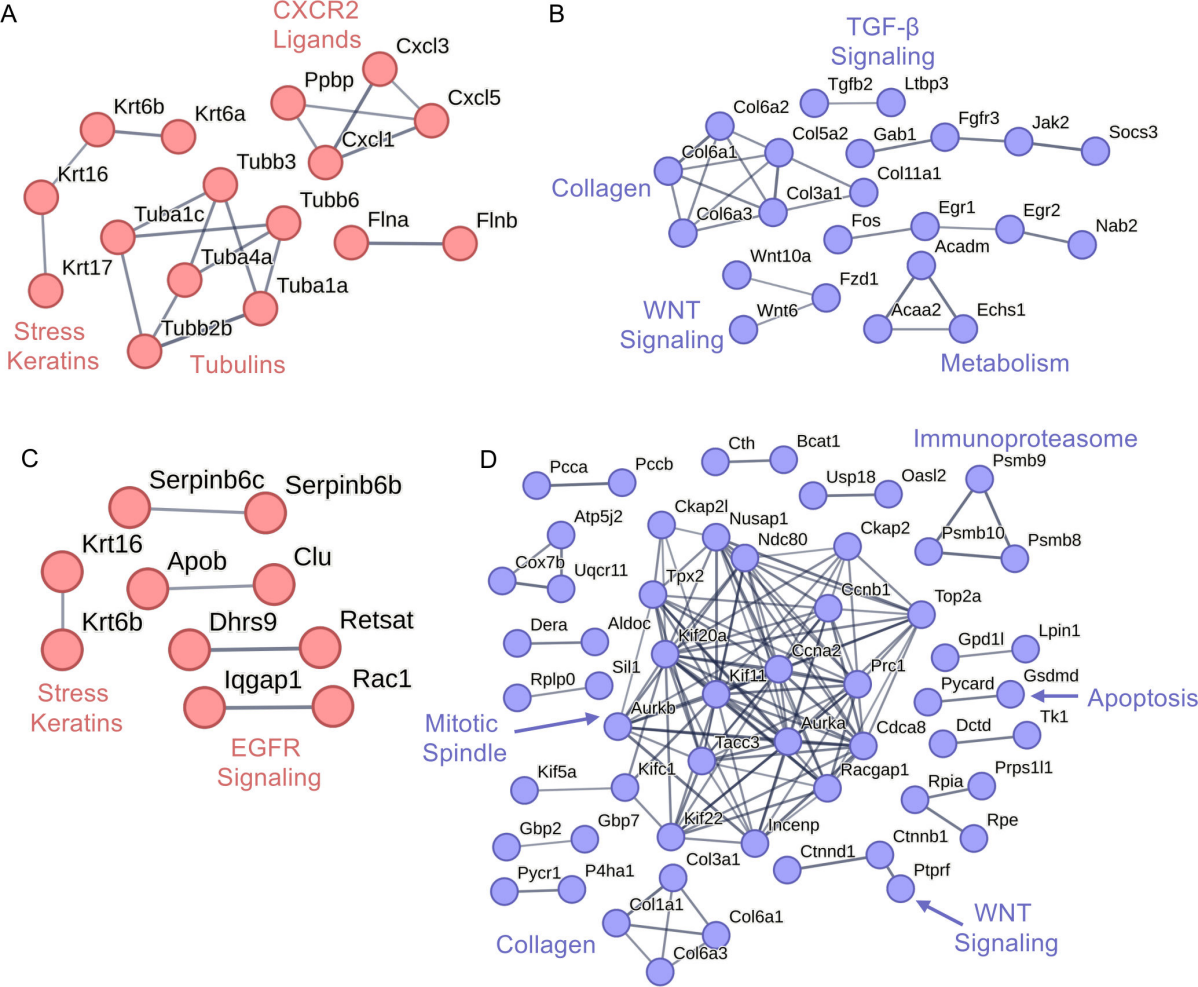
STRING analysis performed on differentially expressed transcripts and proteins. (**A**) Transcripts with log2(FC) > 1 and *P*_adj_ < 0.05 were subjected to STRING analysis. Following the removal of the text mining setting, STRING networks were generated from the transcript list. All upregulated cluster nodes are shown in red. CXCR2 ligands, tubulins, and stress keratin clusters are labeled. (**B**) STRING analysis was performed on transcripts with log2(FC) < −1 and *P*_adj_ < 0.05 were subjected to STRING analysis. Following the removal of text mining interactions, STRING networks were generated from the transcript list. All downregulated cluster nodes are shown in blue. Specific clusters, including TGF-β, collagen, WNT signaling, and metabolism, are labeled. (**C**) STRING analysis was performed on proteins with log2(FC) > 1 and *P*_adj_ < 0.05. Following the removal of text mining interactions, STRING networks were generated from the protein list. All upregulated cluster nodes are shown in red. EGFR signaling and stress keratin clusters are labeled. (**D**) STRING analysis was performed on proteins with log2(FC) < −1 and *P*_adj_ < 0.05. Following the removal of text mining interactions, STRING networks were generated from the protein list. All downregulated cluster nodes are shown in blue. Specific clusters, including immunoproteasome, apoptosis, WNT signaling, collagen, and mitotic spindle, are labeled.

As the genes mentioned above were selected from our analysis, we wanted to determine, in an unbiased manner, the impact of MmuPV1 E7 on cellular signaling cascades and cellular biology. To determine the impact of MmuPV1 E7 on cellular protein-interacting networks, we performed STRING analysis on both our RNA-seq and proteomic analyses. Significantly upregulated [log2(FC) > 1 and *P*_adj_ < 0.05] genes and downregulated [log2(FC) < −1 and *P*_adj_ < 0.05] genes in both our RNA-seq and proteomic data were uploaded to the STRING database (https://string-db.org/). STRING analysis was performed individually on upregulated and downregulated gene sets. To generate interactive networks, STRING analysis settings were set to include the full STRING network, removed text mining as a source for active interaction, and high confidence to minimize less relevant interactions. STRING analysis of our RNA-seq data showed that MmuPV1 E7 increases the expression of CXCR2 ligands, tubulins, and stress keratins (Fig. 2A) and reduces expression of tumor growth factor beta (TGF-β) signaling proteins, collagens, WNT signaling proteins, and metabolism-related proteins (Fig. 2B). We performed an identical analysis on our proteomics data. MmuPV1 E7 increased the abundance of stress keratin and EGF-signaling proteins (Fig. 2C) and reduced the abundance of mitotic spindle, immunoproteasome, collagen, apoptosis, and WNT signaling proteins (Fig. 2D). Our analysis revealed synergism between our RNA-seq and proteomic analyses, particularly in stress keratins, WNT signaling, and collagen genes/proteins. Of particular interest, the increase in levels of stress keratins and CXCR2 ligands and the downregulation of immunoproteasome match observations made during “high-risk” HPV infection and have been linked to activities of “high-risk” HPV E5, E6, and E7 (63, 66–69).

**Fig 3:**
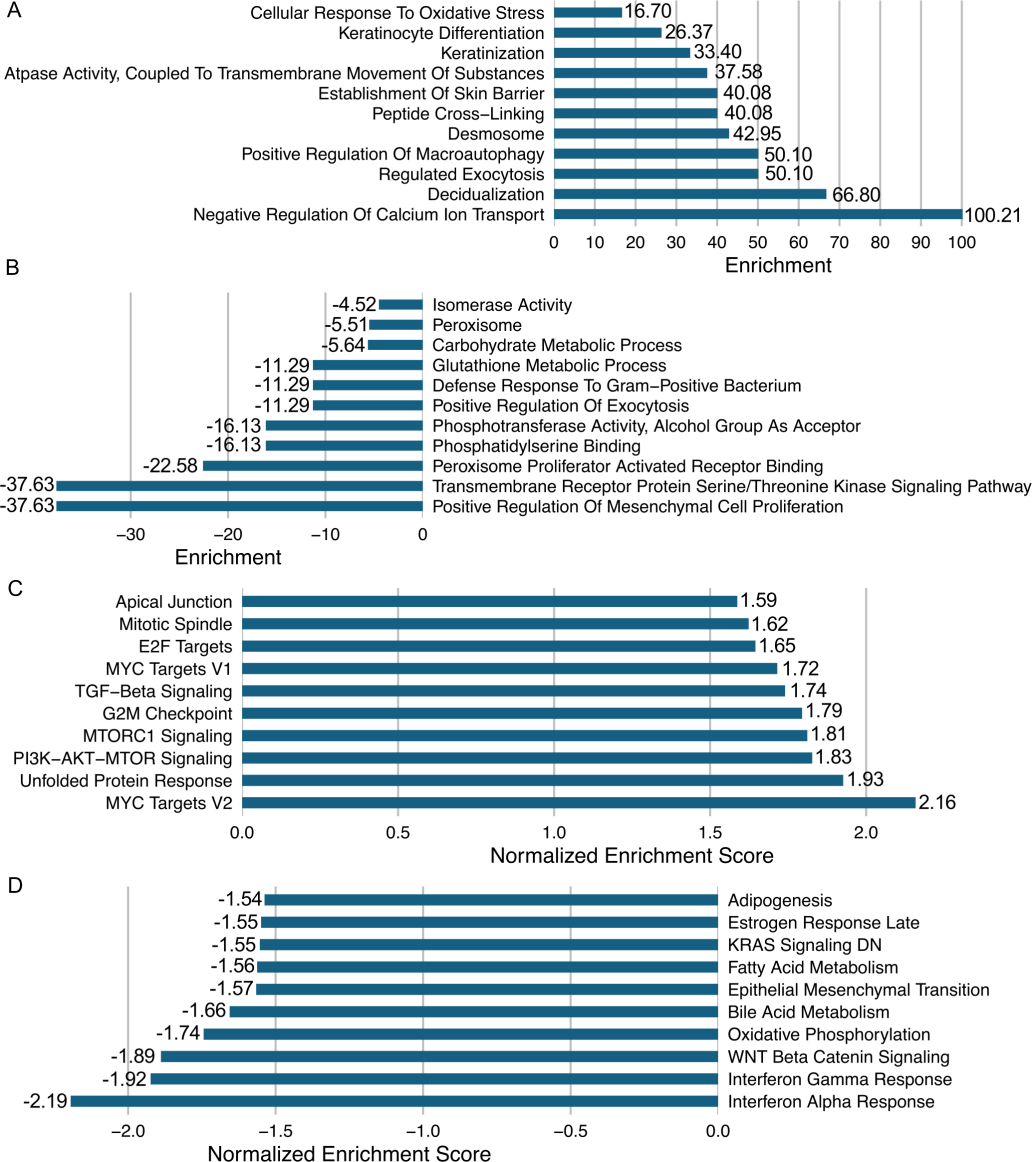
Gene ontology enrichment analysis on proteomic data shows elevation in skin-specific protein gene sets, while gene set enrichment analysis (GSEA) on RNA-seq reveals transcript alterations commonly observed during a papillomavirus infection. (**A**) GO term enrichment was performed on proteomics data specifically on upregulated proteins with log2(FC) > 1 and *P*_adj_ <0.05. The top 11 upregulated gene sets are shown, which have a enrichment score (ES) > 10 and *P*-value <0.05. Enrichment score is shown on the *x*-axis. Gene sets of interest include keratinocyte differentiation and keratinization, with driver proteins being stress keratins. (**B**) GO term enrichment was performed on proteomics data specifically on downregulated proteins with log2(FC) < −1 and *P*_adj_ <0.05. The top 11 downregulated gene sets are shown, which have an ES < −5 and *P*-value <0.05. Enrichment score is shown on the *x*-axis. Gene sets of note include positive regulation of mesenchymal to epithelial transition, carbohydrate metabolic process, and defense responses to gram-positive bacteria (immune related). (**C**) GSEA was performed on RNA-seq data specifically on upregulated transcripts with log2(FC) > 1 and *P*_adj_ <0.05. Analysis was limited to the Hallmark gene sets, which are curated by the Broad Institute. The top 10 upregulated gene sets are shown, with an normalized enrichment score (NES) > 1.5 and false discovery rate (FDR) q-value < 0.05. NES is shown on the *x*-axis. Gene sets of note include “MYC Targets V2,” “PI3K-AKT-MTOR Signaling,” “MTORC1 Signaling,” “G2M Checkpoint,” and “Unfolded Protein Response.” (**D**) GSEA was performed on RNA-seq data specifically on downregulated transcripts with log2(FC) < −1 and *P*_adj_ <0.05. Analysis was limited to the Hallmark gene sets, which are curated by the Broad Institute. The top 10 downregulated gene sets are shown, which have an NES < −1.5 and FDR q-value <0.05. NES is shown on the *x*-axis. Gene sets of note include “Interferon Alpha Response,” “Interferon Gamma Response,” “WNT Beta Catenin Signaling,” and “Epithelial Mesenchymal Transition.”

To determine MmuPV1 E7’s impact on steady-state protein levels on the various signaling pathways in mouse keratinocytes, we utilized gene ontology (GO) analysis as an unbiased analysis to address this question. The GO analysis of our proteomic data showed that MmuPV1 E7 alters proteins associated with various cellular signaling pathways (Fig. 3). MmuPV1 E7 expression correlated with an increase in proteins associated with “Keratinization” and “Establishment of Skin Barrier” (Fig. 3A), which was predominantly associated with the increase in the abundance of stress keratins K16 and K6a. This observation correlated with a decrease in protein abundance of genes associated with “Positive Regulation of Mesenchymal to Epithelial Transition” (Fig. 3B). These data suggest that MmuPV1 E7 appears to promote keratinocyte identity in our mouse keratinocyte culture model system.

To determine if the transcriptional changes we observed in our MmuPV1 E7-expressing cell strains could be tied to specific cellular signaling pathways, we performed gene set enrichment analysis (GSEA) on our RNA-seq analysis, as we had performed in our previous publication, and limited our analysis to the Hallmark gene sets, which are curated by the Broad Institute (57, 70). We observed several signaling pathways that are up- and downregulated in the presence of MmuPV1 E7 compared to our vector control condition (Fig. 3). We observed significant positive enrichment of PI3K (PI3K-AKT-MTOR Signaling) and mTOR signaling (MTORC1 Signaling) in our GSEA (Fig. 3C). We also observed significant positive enrichment with cellular proliferation signaling pathways (G2M Checkpoint) (Fig. 3C). Additionally, we also observed positive enrichment of transcripts associated with TGF-β signaling (TGF Beta Signaling), which included a significant increase in the inhibitory SMAD SMAD7 [log2(FC) = 1.57 and adjusted *P*-value < 0.001) (Fig. 3C). We also observed significantly lower enrichment of immune-related signaling pathways including type 1 and 2 interferon signaling (Interferon Alpha Response and Interferon Gamma Response) (Fig. 3D). We also observed negative enrichment of genes associated with WNT signaling (WNT Beta Catenin Signaling) (Fig. 3D), which is similar to observations made with cutaneous HPV genotypes (71). Consistent with our proteomics analysis, we observed a negative enrichment of epithelial-to-mesenchymal transition genes (Epithelial Mesenchymal Transition) (Fig. 3D), suggesting that MmuPV1 E7 promotes expression of epithelial cell identity. Our collective unbiased analysis of our proteomic and transcriptomic analyses determined that MmuPV1 E7 significantly alters cellular homeostasis on its own and contributes to phenotypes observed during HPV infection.

### MmuPV1 E7 does not increase proliferation rate of mouse keratinocytes

**Fig 4:**
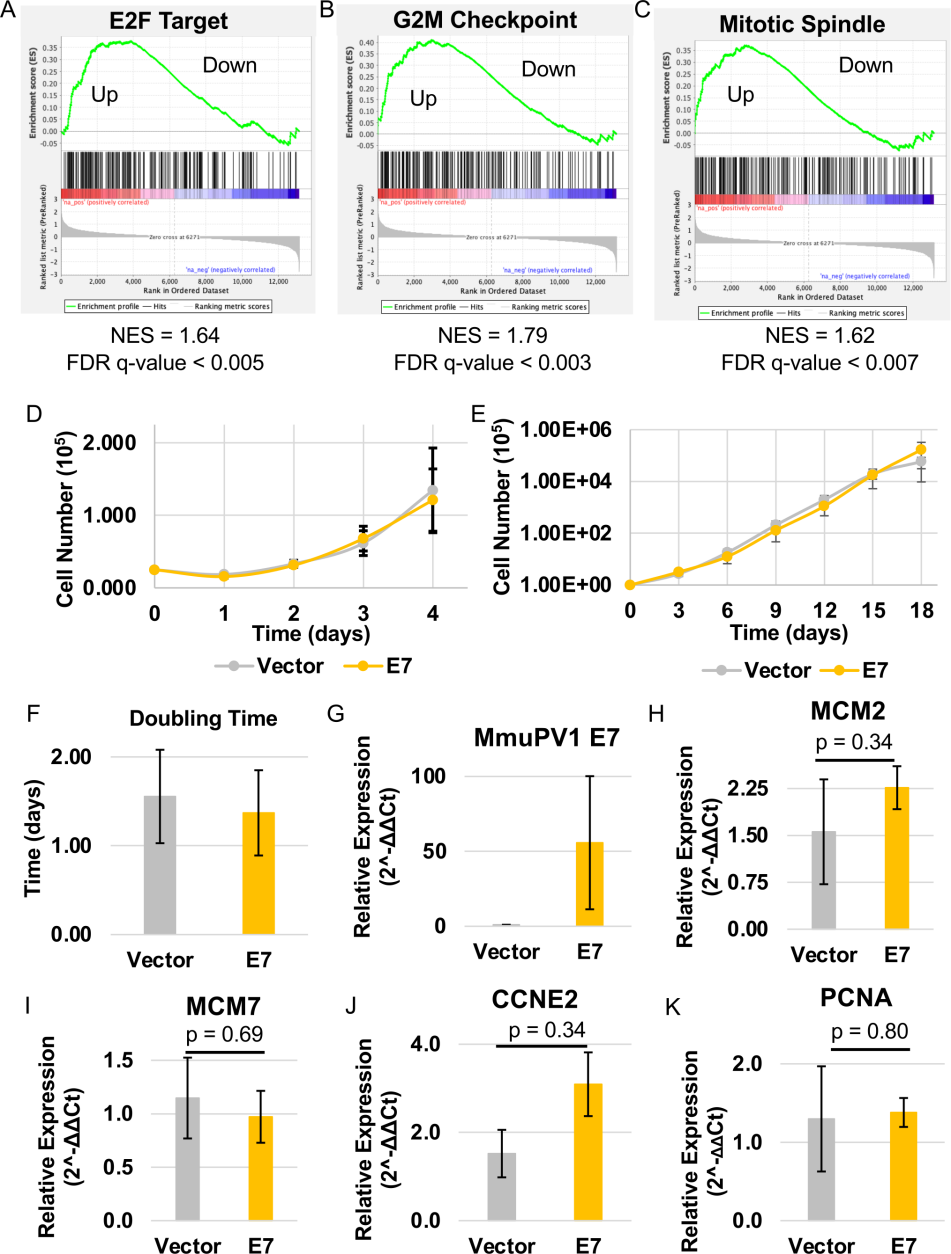
MmuPV1 E7 does not promote increased proliferation and E2F-responsive gene expression. GSEA plots for “E2F Target” (**A**), “G2M Checkpoint” (**B**), and “Mitotic Spindle” (**C**) gene sets. Normalized enrichment score (NES) and false discovery rate (FDR) q-values are shown. “Up” indicates the portion of plot with upregulated transcripts, and “Down” indicates the portion of the plot with downregulated transcripts. (**D**) Short-term growth analysis of MmuPV1 E7-expressing and vector control MKs is shown. A total of 0.25 × 10^5^ cells were plated into four wells of a six-well plate. Cells were counted daily for 4 days following plating of MKs. The average at each time point is shown, and standard error is shown. (**E**) Long-term growth analysis of MmuPV1 E7-expressing or vector control MKs is shown. A total of 1 × 10^5^ cells were plated into a 60 mm dish. Cells were counted and replated at 1 × 10^5^ cells every 3 days for an 18 day period. Average cumulative growth is shown over an 18 day period. (**F**) Doubling time was calculated for each cell strain by calculating the growth rate of each cell strain. The average doubling time with standard error is shown. (**G**) qRT-PCR was performed on cDNA generated from RNA isolated from MmuPV1 E7-expressing and vector control MKs using primers for MmuPV1 E7. Additionally, qRT-PCR was performed on cDNA using primers targeting MCM2 (**H**), MCM7 (**I**), CCNE2 (**J**), and PCNA (**K**). ΔΔCt was calculated with average and standard error shown. The Wilcoxon rank-sum test was performed, and *P*-values are shown.

A traditional hallmark of HPV E7 oncoproteins is increasing the expression of E2F transcription factor target genes by inhibiting the tumor suppressor pRB (25). We have previously shown that MmuPV1 E6 upregulates E2F target gene transcription in mouse keratinocytes (57). However, we observed a weak but significant positive enrichment of proliferation genes in MmuPV1 E7 expressing MKs (Fig. 4A through C). The strongest enrichment is in the G2M checkpoint gene set (G2M Checkpoint) (Fig. 4B). Interestingly, we did observe a weak but significant positive enrichment of mitotic spindle (Mitotic Spindle) genes transcriptionally, but our STRING analysis of our proteomics data shows a cluster of mitotic spindle proteins that are decreased in our proteomic analysis (Fig. 2D). To determine the impact of the increased enrichment of proliferation-associated genes in our RNA-seq analysis (Fig. 4A through C), we performed growth curve analysis of MmuPV1 E7-expressing MKs compared to vector control MKs (Fig. 4D and E) for cellular proliferation. We used our short- (4 days) and long-term (18 days) growth experiments that we previously published studying MmuPV1 E6-expressing MKs (57). We observed no significant difference in the growth rate between our MmuPV1 E7 and vector control MKs in either experiment (Fig. 4D and E). Using this data, we calculated the doubling time of MmuPV1 E7-expressing and vector control MKs and observed no difference (Fig. 4F). Using qRT-PCR, we confirmed expression of E7 in our MmuPV1 E7-expressing MKs with significantly lower signal in our vector control MKs (Fig. 4G). To validate the increased expression of proliferation-associated genes, we performed qRT-PCR using validated primers for E2F-responsive genes that are activated by HPV E7 (MCM2, MCM7, CCNE2, and PCNA) (Fig. 4H through K) (53, 57). We did not observe a significant increase in the expression of the E2F-responsive genes (Fig. 4H through K). These results are consistent with previously published results (53). Therefore, our RNA-seq results may suggest a mild positive enrichment of proliferation-associated genes in our MmuPV1 E7-expressing MKs, but the alteration in proliferation genes did not correlate with changes in the proliferation and growth capacity of mouse keratinocytes.

### MmuPV1 E7 increases the expression of stress keratins in mouse keratinocytes

**Fig 5:**
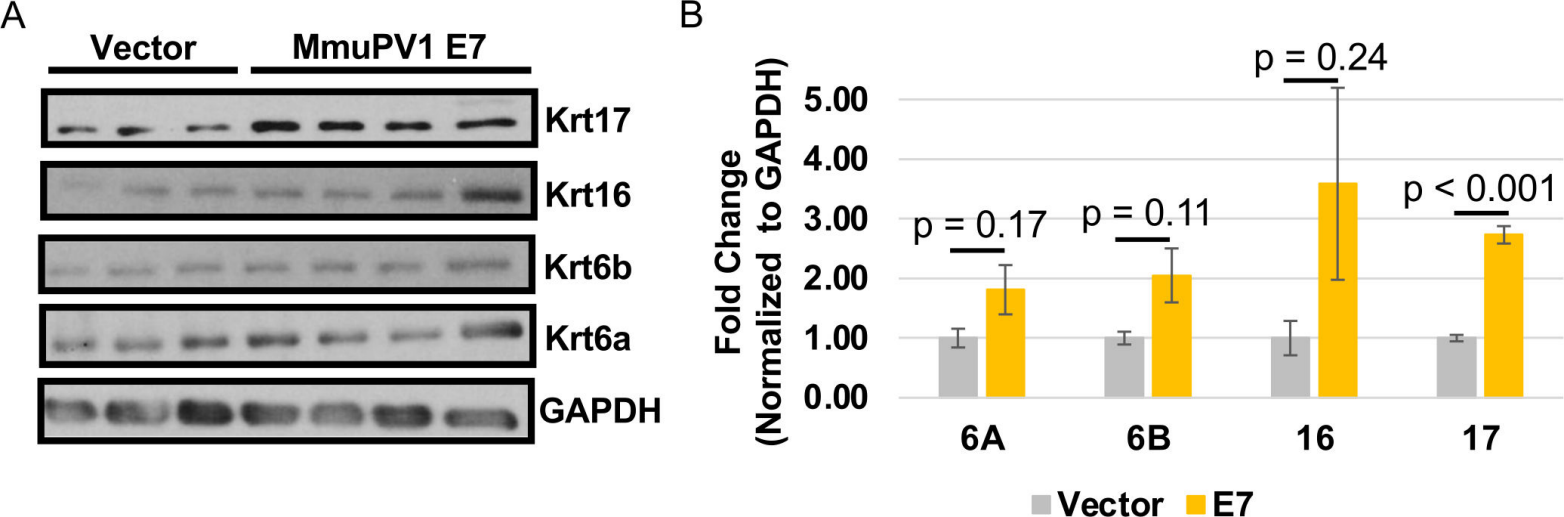
MmuPV1 E7 promotes expression of the stress keratin K17. (**A**) Western blot analysis of MmuPV1 E7-expressing MKs was performed on protein extracts isolated from MKs. Antibodies against keratin 17 (Krt17), keratin 16 (Krt16), keratin 6a/6b (Krt6a/Krt6b), and GAPDH were used in this analysis. Western blot analysis was quantified using ImageJ (**B**) and normalized to GAPDH. Average of biological replicates and standard error is shown. The Wilcoxon rank-sum test was performed on analysis, with *P*-values shown. Analysis was performed on at least biological triplicates, meaning three MK cell strains isolated from three different mice.

In our RNA-seq analysis, we observed a significant increase in the transcription of stress keratin genes (Krt6a, Krt6b, Krt16, and Krt17) (Table 2). Stress keratins have been previously shown to be increased during MmuPV1 infection in immunocompetent FVB/N mice, with Krt17 playing a key role in inhibiting T cell response (59). However, the mechanism by which MmuPV1 promotes expression of stress keratins, including Krt17, remains unknown. Additionally, we did detect two of the stress keratins in our proteomics data (Krt6b and Krt16), and only Krt16 showed a significant increase in protein abundance (Table 2). We did not detect Krt6a and Krt17 in our proteomic analysis. To validate the increase in expression of the various stress keratins, we performed immunoblot analysis on vector control and MmuPV1 E7-expressing MKs (Fig. 5). We observed a significant increase in the steady-state levels of Krt17 in MmuPV1 E7-expressing MKs compared to the vector control MKs (Fig. 5). The other stress keratins were slightly but not significantly increased in the MmuPV1 E7-expressing MKs compared to vector control MKs (Fig. 5). These data suggest that MmuPV1 E7 increases the expression of Krt17 and may contribute to elevated expression of the other stress keratins during MmuPV1 infection. Thus, our data provide evidence that MmuPV1 E7 contributes to immune evasion through promoting K17 expression, which has previously been shown to decrease T cell recruitment to MmuPV1-infected warts (59).

**TABLE 2:** Results from RNA-seq and proteomic analyses for stress keratin genes^*a*^

Gene	RNA-seq			Proteomics		
	Log2(FC)	Fold change	*P*-value	Log2(FC)	Fold change	*P* value
Krt6a	1.41	2.65	0.0071	ND	ND	ND
Krt6b	1.69	3.22	0.0186	1.63	3.10	0.100
Krt16	2.83	7.12	0.0017	1.37	2.58	0.011
Krt17	1.60	3.04	0.0105	ND	ND	ND

^*a*^ The log2(FC), fold change, and *P*-value (adjusted) are shown for each stress keratin gene, including Krt6a, Krt6b, Krt16, and Krt17. ND is not detected.

### MmuPV1 E7 promotes mTOR signaling in mouse keratinocytes

**Fig 6:**
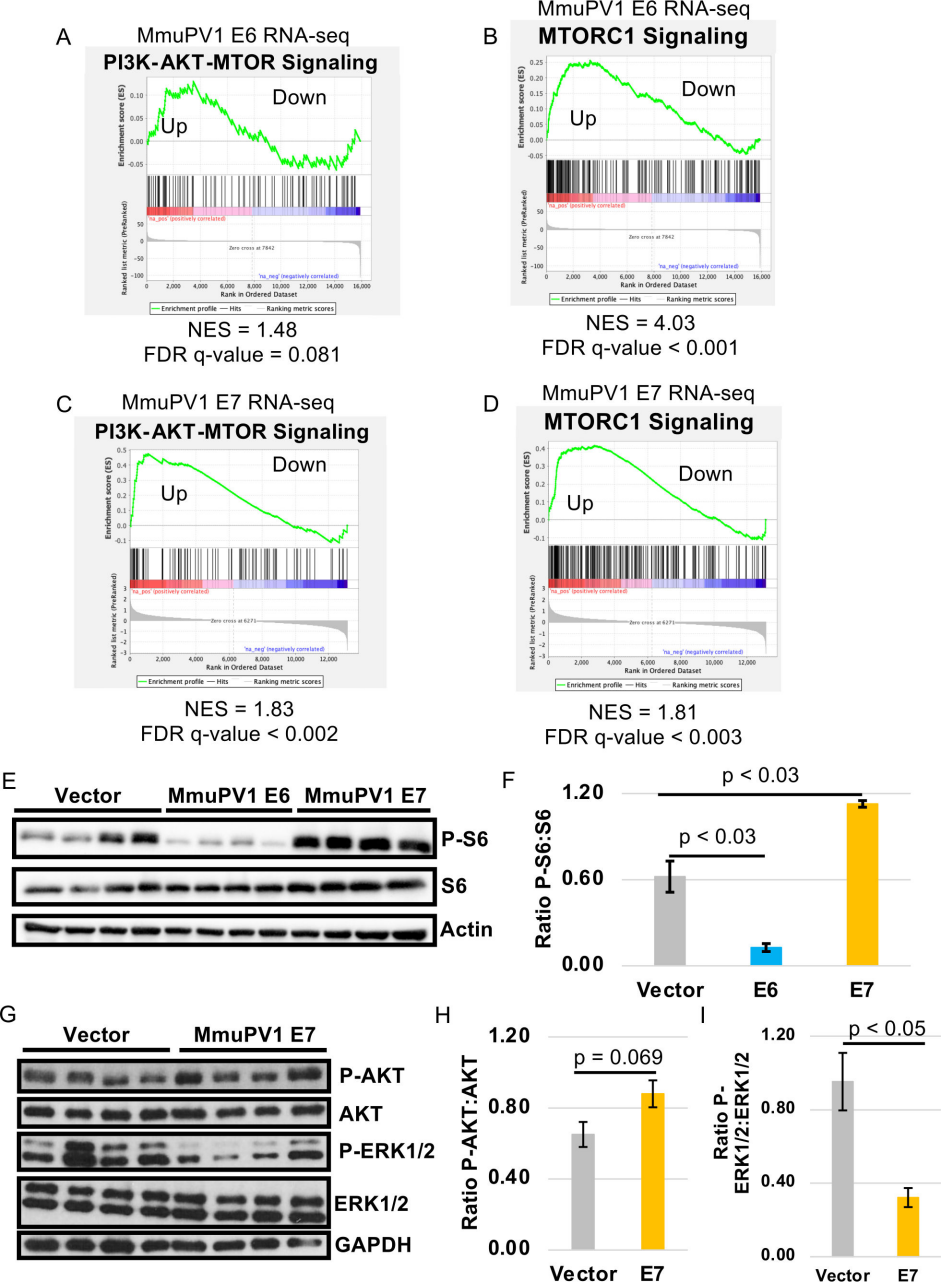
MmuPV1 E7 promotes mTOR and PI3K signaling in mouse keratinocytes. GSEA of previously published RNA-seq on MmuPV1 E6-expressing compared to vector control MKs showing GSEA plots for “PI3K-AKT-MTOR Signaling” (**A**) and “MTORC1 Signaling” (**B**) (57). GSEA on MmuPV1 E7-expressing compared to vector control MKs showing GSEA plots for “PI3K-AKT-MTOR Signaling” (**C**) and “MTORC1 Signaling” (**D**). Normalized enrichment scores (NES) and false discovery rate (FDR) q-values are shown. In this analysis, “Up” indicates regions of GSEA plots with upregulated genes, and “Down” indicates regions in GSEA plots with downregulated genes. (**E**) Western blot analysis on protein extracts isolated from MmuPV1 E7-expressing, MmuPV1 E6-expressing, and vector control MKs. Western blots were performed using antibodies against P-S6, S6 kinase, and actin. (**F**) Quantification was performed using ImageJ, and the ratio of P-S6:S6 was determined. Average ratios and standard errors are shown. Wilcoxon rank-sum test was performed on analysis. (**G**) Western blot analysis was performed on protein extracts isolated from vector and MmuPV1 E7-expressing MKs using antibodies against phosphorylated AKT (P-AKT), total AKT, phosphorylated ERK1/2 (P-ERK1/2), total ERK 1/2, and GAPDH. Quantification of western blot analysis is shown comparing the ratio of P-AKT:AKT (**H**) and P-ERK1/2:ERK1/2 (**I**). Average ratios and standard error are shown. Wilcoxon rank-sum test was performed, and *P*-values are shown.

Previous work has found that HPV and MmuPV1 both elevate EGFR and mTOR signaling in mice (66, 67, 72–74). For “high-risk” HPVs, the literature has shown that E5 and E6 contribute to promotion of EGFR and mTOR signaling in human keratinocytes (66, 67, 72–74). In our previously published MmuPV1 E6 RNA-seq analysis, we found that PI3K signaling (PI3K-AKT-MTOR Signaling) was not significantly positively enriched in our analysis (Fig. 6A) (57). We did see a significant positive enrichment of genes associated with mTOR signaling (MTORC1 Signaling) (Fig. 6B) (57). These results would suggest potential overlap between MmuPV1 E6 and HPV E6. However, we found that several of the genes that drive this positive enrichment are also associated with proliferation, consistent with our previous results (57). We did observe that genes associated with PI3K (PI3K-AKT-MTOR Signaling) and mTOR (MTORC1 Signaling) were both significantly positively enriched in our MmuPV1 E7-expressing MKs compared to vector control MKs (Fig. 6C and D). To determine whether MmuPV1 E6, MmuPV1 E7, or both activate the PI3K-AKT-mTOR signaling cascade, we performed immunoblot analysis for a downstream marker for activation of this signaling cascade, phosphorylated S6 kinase (P-S6), comparing MmuPV1 E6-expressing, MmuPV1 E7-expressing, and vector control MKs. Our immunoblot analysis showed that MmuPV1 E7 leads to a significant increase in the steady-state levels of P-S6 kinase relative to total S6 kinase (Fig. 6E and F). In contrast, MmuPV1 E6 expression led to a significant decrease in the steady-state levels of P-S6 kinase relative to total S6 kinase (Fig. 6E and F). These data would suggest that MmuPV1 E7, and not MmuPV1 E6, promotes activation of downstream targets of PI3K and mTOR signaling. Previous work has linked activation of S6 kinase and mTOR signaling through EGFR activation during HPV and MmuPV1 infection (67). To determine the impact of MmuPV1 E7 on EGFR signaling in mouse keratinocytes, we performed immunoblot analysis for phosphorylation of AKT (S473) and ERK1/2 (T202/Y204), which are known EGFR signaling phosphorylation sites. We observed a slight but non-significant increase in the steady-state ratio of phosphorylated AKT to total AKT in our MmuPV1 E7-expressing MKs (Fig. 6G and H). We did observe a significant decrease in the levels of phosphorylated ERK1/2 relative to total ERK1/2 in our MmuPV1 E7-expressing MKs (Fig. 6G and I). We observed no difference in the total levels of ERK1/2 (Fig. 6G). Our collective data suggest that MmuPV1 E7 promotes mTOR signaling (elevated P-S6), but upstream markers of EGFR signaling associated with mTOR signaling are not significantly increased.

### MmuPV1 E7 potentiates EGF signaling in mouse keratinocytes

**Fig 7:**
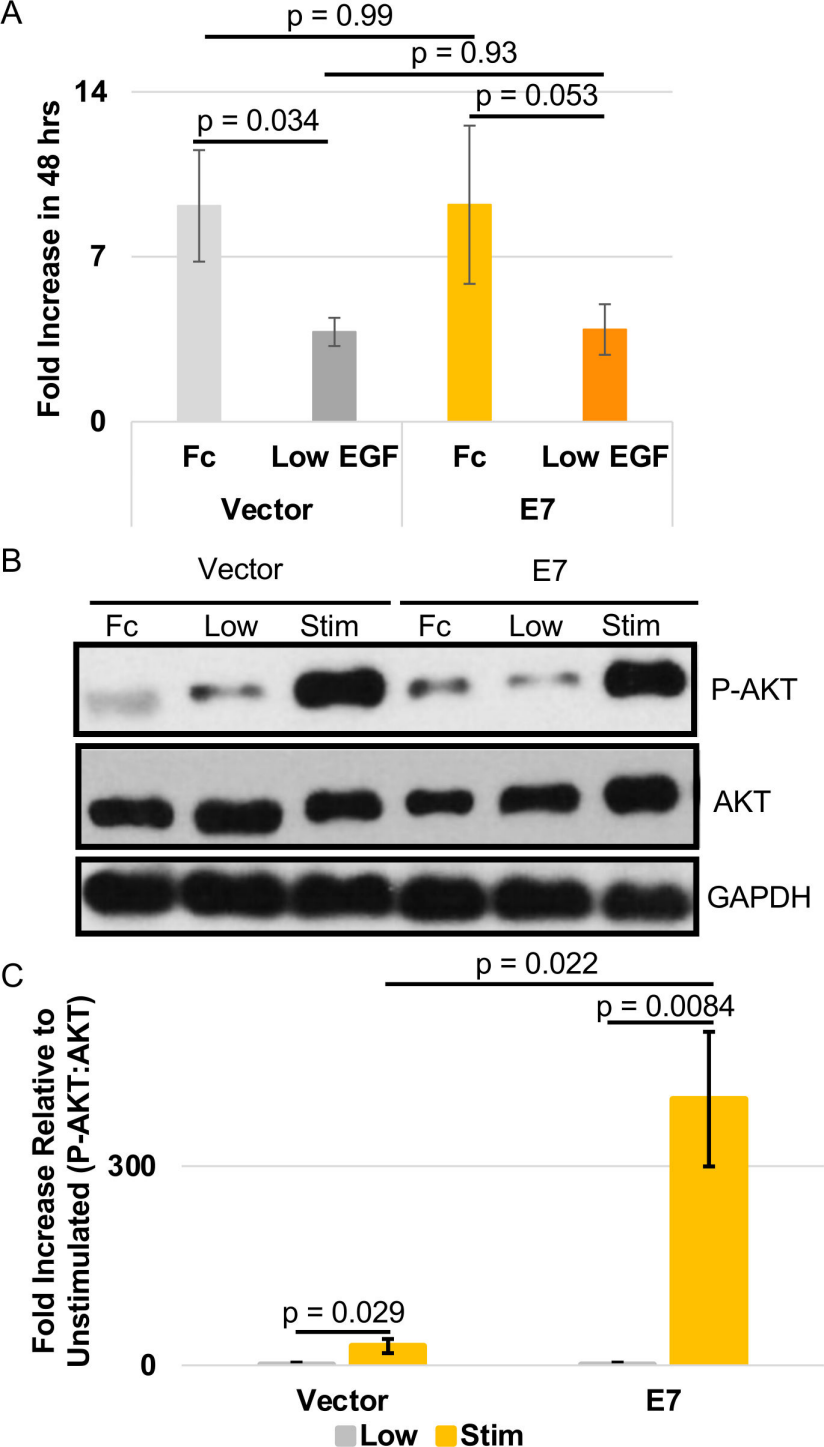
MmuPV1 E7-expressing mouse keratinocytes have increased response to EGF stimulation. (**A**) MmuPV1 E7-expressing and vector control MKs were grown in either F-media (Fc) or EGF-low (low) F-media (1% FBS and no exogenous EGF added to media). MKs were plated at 0.5 × 10^5^ and then grown for 48 hours in either medium condition. Cells were counted at 48 hours, and fold increase in cell number was determined at that time point. Average fold increase and standard error are shown. Wilcoxon rank-sum test was performed, and *P*-values are shown. (**B**) EGF stimulation experiments comparing vector control and MmuPV1 E7-expressing MKs. Cells were grown to near confluency in F-media (Fc) and switched to EGF-low (low) F-media approximately 18 hours prior to stimulation. Protein extract was collected at this time point. After stabilization in EGF-low F-media, cells were either mock-treated or treated with 10 ng/mL of EGF (stim) for 5 minutes. Cells were lysed in radioimmunoprecipitation assay (RIPA) buffer to make protein extracts. (**B**) Western blot analysis was performed in protein extracts using antibodies against phosphorylated AKT (P-AKT), total AKT, and GAPDH. Representative western blot of biological triplicate is shown. (**C**) Western blots were quantified using ImageJ, and the ratio of P-AKT:AKT was determined. Average fold induction comparing treated to untreated with standard error is shown. The Wilcoxon rank-sum test was performed, with *P*-value shown.

As stated above, we did not observe a significant increase in the steady-state abundance of markers of EGFR signaling with a trend in promoting phosphorylation of AKT. However, the mouse keratinocytes were grown and maintained in F-media containing ROCK inhibitor. F-media contains exogenous EGF and 5% serum (fetal bovine serum [FBS]). Therefore, the mouse keratinocytes are maintained in a tonic EGFR signaling environment, which could mask any phenotypic changes in EGFR signaling promoted by MmuPV1 E7. To address whether F-media is masking potential phenotypes, we grew mouse keratinocytes in EGF-low F-media by removing the exogenous EGF and lowering the FBS percentage from 5% to 1% (EGF-low). First, we performed a growth analysis of these cells in the EGF-low environment to determine if MmuPV1 E7 increases the proliferation of mouse keratinocytes in this environment. We plated cells and counted the keratinocytes 18 hours later to determine the number of cells plated. The media on the cells was changed to either F-media (without ROCK inhibitor) or EGF-low F-media. After 48 hours, we counted the cells and determined the fold change in growth. We observed no difference in the fold increase in the MmuPV1 E7-expressing MKs compared to the vector control MKs in the EGF-low F-media (Fig. 7A). As expected, we also observed no difference in the growth rate of MmuPV1 E7 mouse keratinocytes compared to vector control mouse keratinocytes in F-media (Fig. 7A). We did observe reduced growth in both MmuPV1 E7-expressing and vector control MKs when EGF levels were minimal (Fig. 7A). These observations suggest that MmuPV1 E7 does not promote the growth of mouse keratinocytes in an EGF-restrictive environment.

While MmuPV1 E7 may not promote the growth of mouse keratinocytes when EGF is restricted, we were interested in determining whether MmuPV1 E7 potentiated EGFR signaling when EGF was added back to the cell culture media. To do this, we grew our mouse keratinocytes in F-media. When the cells were 24 hours prior to confluency, we washed them 3× with phosphate-buffered saline (PBS) to remove F-media and grew cells in EGF-low F-media for 18 hours to allow the EGFR signaling to stabilize in the MKs. Following stabilization in EGF-low F-media, we removed the feeders using trypsin the following morning. We stimulated vector control or MmuPV1 E7-expressing MKs with 10 ng/mL EGF for 5 minutes. After stimulation, cells were washed 2× with cold PBS to slow signaling events and collected in RIPA buffer. Lysates were subjected to immunoblot analysis using antibodies against AKT, P-AKT, and GAPDH. We observed an increase in P-AKT levels in our vector control cells following stimulation with EGF (Fig. 7B). Similarly, we also observed an increase in P-AKT in the MmuPV1 E7-expressing MKs with no consistent impact on total AKT levels (Fig. 7B; Fig. S1A). To determine the impact of MmuPV1 E7 on potentiating EGF signaling in mouse keratinocytes, we quantified the western blot by normalizing the levels of P-AKT to total AKT in the EGF-low and the EGF-stim groups. We observed that MmuPV1 E7 increased the ability of mouse keratinocytes to respond to EGF stimulation following 18 hours of growth in minimal EGF media (Fig. 7C). These data suggest that MmuPV1 E7 potentiates EGFR signaling, which likely contributes to increased mTOR signaling in mouse keratinocytes.

### Interactive network analysis shows connections between MmuPV1 E7, E7 interacting partners, and mTOR signaling

**Fig 8:**
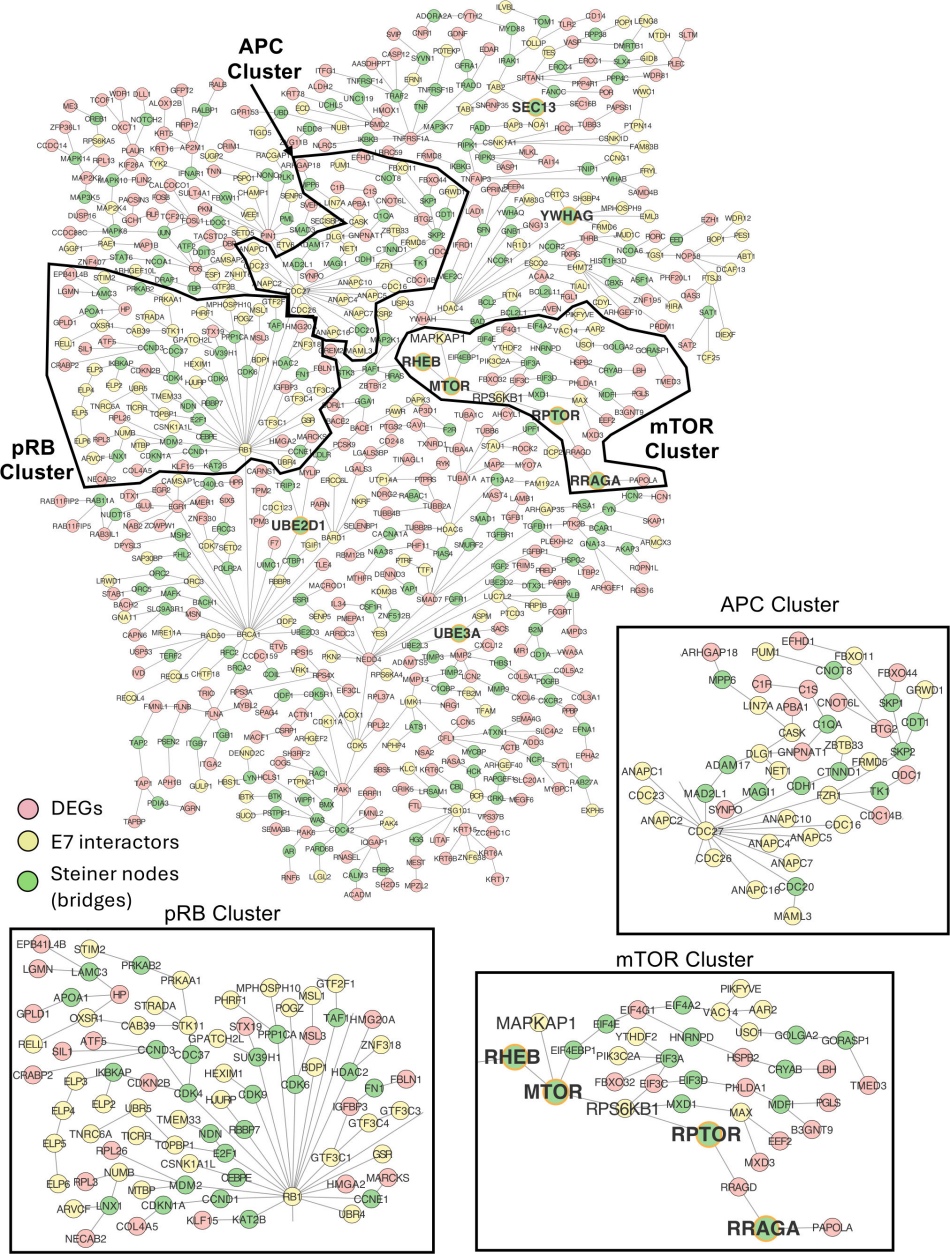
Network analysis of MmuPV1 E7-expressing mouse keratinocytes shows Steiner node enrichment in mTOR signaling. MmuPV1 E7 differentially expressed genes in our RNA-seq analysis and affinity-purification mass spectrometry (interactome) data were connected using a prize-collecting Steiner forest algorithm. The E7 interacting partners (yellow) were enriched in cell cycle and cell division, whereas the differentially expressed genes (pink) were enriched for cell differentiation and motility. The Steiner nodes (green) that bridge between DEGs and interactors were enriched in mTORC1 signaling, cell division, anoikis, T cell-mediated immunity, and chromatin remodeling. Differentially expressed genes were defined as all transcripts with adjusted *P*-value <0.05. mTOR signaling, APC, and pRB clusters are magnified.

An interactome network analysis was performed to connect the differentially expressed genes (DEGs) in our RNA-seq analysis and the interactors of MmuPV1 E7 identified by the Munger lab (Tufts University) (53). The prize-collecting Steiner forest (PCSF) algorithm was used to find the pathways connecting the interactors and DEGs through the minimum number of protein-protein interactions and bridging nodes (also known as Steiner nodes). A key feature that we observed in our network analysis was an enrichment of cell division and cell cycle proteins in the MmuPV1 E7 interactome data (yellow nodes) (Fig. 8). On the other hand, the DEGs were enriched for cell differentiation, cell motility, neurogenesis, and programmed cell death (pink nodes) (Fig. 8). Interestingly, the Gray Steiner (bridging) nodes in this analysis were enriched for genes associated with cell division, anoikis, mTORC1 signaling, T cell-mediated immunity, and chromatin remodeling (green nodes) (Fig. 8). The enrichment in mTOR signaling among the bridging nodes suggests that MmuPV1 E7 may directly impact mTOR signaling in mouse keratinocytes through its interacting partners instead of an indirect mechanism (bolded nodes). The network map revealed several distinct clusters within the analysis, including clusters focused on mTOR signaling, anaphase-promoting complex (APC), and pRB (Fig. 8). The pRB cluster is particularly interesting, as there are several Steiner nodes that bridge E7 interacting partners, including CDK6, CDK9, CCNE1, and E2F1. The mTOR signaling cluster includes the Steiner nodes MTOR and RPTOR connected to the E7 interacting partners RPS6KB1 and MAPKAP1, providing evidence that MmuPV1 E7 may directly impact mTOR signaling through its interacting partners. Moreover, this connection between MmuPV1 E7 interacting partners and DEGs in MmuPV1 E7-expressing mouse keratinocytes also highlights the concordance among our transcriptomic, proteomic, and confirmation studies.

## DISCUSSION

In this study, we complement our previous work on understanding the role of MmuPV1 E6 in promoting phenotypes associated with the hallmarks of cancers by performing multiomics analysis of MmuPV1 E7-expressing MKs (57). Our collective observations suggest that MmuPV1 E7 does alter cellular homeostasis and promotes phenotypes commonly observed in cancer-causing “high-risk” HPV infection. Previous studies of MmuPV1 E7 have identified at least two interacting partners, pRB and PTPN14, that are shared between HPV E7 and MmuPV1 E7 (53, 54). However, the impact of these interactions on cellular homeostasis is understudied, and studies were limited to phenotypes associated with biological impacts of the “high-risk” HPV E7 oncogenes. To remedy this gap in knowledge, we performed a series of omics analyses examining the impact of MmuPV1 E7 on keratinocyte homeostasis. We utilized our ability to perform proteomic (quantitative mass spec) and transcriptomic (RNA-seq) analyses on mouse keratinocytes that stably express MmuPV1 E7 compared to vector control MKs. MmuPV1 E7 caused significant changes to the cellular transcriptome and proteome, including several changes that are implicated in HPV-associated cancers, including cervical and head and neck cancer (Fig. 1). Notable genes include Ptgs2 (up), Hmox1 (up), and Arhgap18 (down) (60–63, 75, 76). Importantly, little is known about the impact of HPV infection on these genes, and it remains unknown if the alterations of Ptgs2, Hmox1, and Arhgap18 are connected to activities of HPV E6 or E7. These observations raise new questions about how MmuPV1 E7 promotes cellular alterations that are related to “high-risk” HPV infections and could lead to the identification of new activities of MmuPV1 and HPV E7 that could be implicated in HPV-associated mucosal cancers and infections.

Overall, MmuPV1 E7 had a smaller phenotype compared to that of MmuPV1 E6 (Table 1). We also found that MmuPV1 E7 had a smaller impact on the differential expression of genes and proteins in our transcriptomic and proteomic analyses and in our GSEA or GO analysis (Fig. 6A through D). We did find that MmuPV1 E7 is expressed at a much lower level compared to MmuPV1 E6 in the RNA-seq analysis (data not shown). This observation suggests that high levels of MmuPV1 E7 could be toxic to cells, and the lower-level expression is what was selected for in our mouse keratinocyte system. However, we did find that our RNA-seq and proteomic analyses were consistent for genes that were detected in both analyses. We were able to determine that 35 genes/proteins were differentially expressed in both of our analyses. Of those 35, 34 genes/proteins show similar results in both the RNA-seq and proteomic analyses. The sole gene/protein that was divergent was Cth (cystathionine gamma-lyase), which showed increased gene expression but reduced protein levels. This would suggest that MmuPV1 E7 promotes reduced steady-state levels of the protein, and future studies could address this difference. Interestingly, two of our most differentially expressed genes/proteins—Hmox1 and Ptgs2—showed an increase in gene expression and protein abundance in our analysis, thus further strengthening the need for future studies to determine the impact of MmuPV1 E7 and HPV E6/E7 on their expression. In addition to these changes in transcription and protein abundance, we found convergence in our GO analysis and GSEA. Specifically, we observed similar enrichment in gene sets that share biological properties. Examples of this include the increases in “Cellular Response to Oxidative Stress” and “Desmosome” in GO analysis and “MTORC1 Signaling” and “Apical Junction” in GSEA, respectively. We also observed biologically similar pathways being negatively enriched in our analysis. Examples of shared negatively enriched pathways include “Carbohydrate Metabolism” and “Defense Response to Gram-Positive Bacterium” in GO analysis and “Oxidative Phosphorylation” and “Interferon Alpha/Gamma Response” in GSEA, respectively. These results suggest that while MmuPV1 E7 may have a smaller impact on cellular homeostasis, there is consistency in the alteration of biological processes both transcriptionally and at the protein level.

It is important to note that we are studying MmuPV1 E7 in isolation and that both MmuPV1 E6 and E7 are required for pathogenesis *in vivo* (52, 53). The interplay between MmuPV1 E7 and E6 remains understudied. Future studies are needed to address the interplay between MmuPV1 E6 and E7 and how they promote the activities of one another. Additionally, there could be cellular alterations that are promoted only in the presence of both MmuPV1 E6 and E7, which have been missed in our studies on MmuPV1 E6 and E7 individually (57). Potential studies could perform transcriptomic and proteomic analyses on MKs that co-express MmuPV1 E6 and E7 to address this question. Additionally, future studies should include examination of the impact of MmuPV1 E6 and E7 co-expression in the context of the entire MmuPV1 genome in MKs to better reflect what is occurring in MmuPV1-infected mice.

Our studies further strengthened the argument that MmuPV1 E7 is not the major driver of proliferation in mouse keratinocytes (Fig. 6) (53). We did observe slight increases in expression of genes related to proliferation in our GSEA, including a positive enrichment of “E2F-Responsive Genes,” “G2M Checkpoint,” and “Mitotic Spindle” (Fig. 6). However, we did not observe a significant increase in the proliferation capacity of MmuPV1 E7-expressing MKs nor an increase in expression of classic E2F-responsive genes. These data suggest that MmuPV1 E7 may promote expression of proliferation genes using an alternative mechanism outside of E2F activation, and further studies are needed to address this observation. Interestingly, STRING analysis of our proteomics data revealed that there is a negative enrichment of several proteins in the mitotic spindle complex, which is in contrast to our RNA-seq analysis, which shows an enrichment of mitotic spindle-associated gene expression. This is the first case of a difference between our transcriptomic and proteomic analyses. However, MmuPV1 E7 does interact with several APC proteins, which were identified in the affinity purification/mass spectrometry analysis performed by the Munger lab (Tufts University) (Fig. 8) (53). This observation suggests that MmuPV1 E7 may impact the protein abundance of the APC complex and other cellular proteins that interact with this complex instead of acting on the transcription of these proteins. The interaction between MmuPV1 E7 and APC complex proteins has yet to be confirmed and/or verified in the appropriate cell lines. Further studies are needed to determine the impact of these interactions in phenotypes associated with MmuPV1 E7 expression.

The impact of MmuPV1 E7’s interaction with pRB still remains unclear, and our study further strengthens the argument that MmuPV1 E7’s interaction with pRB does not promote traditional phenotypes. However, the interactome network analysis did reveal a distinct pRB cluster of DEGs, E7 interacting partners, and Steiner nodes with pRB appearing as the central node in this cluster (Fig. 8). Of particular interest are the Steiner ray nodes that bridge pRB and a subset of DEGs and other MmuPV1 E7 interacting partners. Steiner nodes of interest include PPP1CA (PP1 catalytic subunit), CDK6, CDK9, CCNE1 (cyclin E), CCND1 (cyclin D1), CDKN1A (p16), and E2F1. The E2F1 Steiner node does appear to connect pRB with TOPBP1 (E7 interacting protein) in our analysis. This is particularly interesting, as TOPBP1 has been shown to be required for genome replication and viral E2 activity (77–79). This observation suggests a potential role for MmuPV1 E7 in viral genome replication and maintenance potentially through its pRB interaction. In addition to this, we do not observe any other E2F transcription factor within this cluster, which potentially suggests that there is E2F1 specificity in the MmuPV1 E7-pRB interaction. The literature has shown that pRB does have a specific interaction with E2F1 outside of the other E2F transcription factors (80–82). The specific interaction between pRB and E2F1 has been shown to be a non-canonical activity of pRB and plays a role in EZH2 activity and DNA damage (80–83). This result provides more evidence that MmuPV1 E7’s interaction with pRB does appear to play a distinct role outside of the traditional pRB-E2F axis. Further studies are needed to better address these observations and characterize the role of MmuPV1 E7’s interaction with pRB.

Our proteomic and transcriptomic analysis did reveal that MmuPV1 E7 promotes a number of phenotypes that are associated with modulation of the immune response, including repression of the type 1 and 2 interferon response (Fig. 3), increased expression of CXCR2 ligands (Fig. 2), and increased expression of stress keratins, in particular K17 (Fig. 5) (59, 69, 84–91). All of these phenotypes are well characterized for “high-risk” HPV infection and, to a lesser extent, cutaneous and “low-risk” HPV infections (84–90). The increase in CXCR2 ligand expression was observed in the HPV16 E6 and E7 transgenic animal model system and was linked to HPV16 E7 expression (69). Additionally, increased K17 expression has been observed in the HPV16 transgenic animal model system and during an MmuPV1 infection of immunocompetent mice (59, 91). The increase in K17 expression is linked to repression of the T cell response and is a key mechanism by which papillomavirus infection promotes immune evasion *in vivo* (59). We were able to confirm that MmuPV1 E7 expression does increase the abundance of K17 in mouse keratinocytes transcriptionally (RNA-seq) (Table 2) and at the protein level (Fig. 5) (59). We still do not understand the mechanism by which MmuPV1 E7 may promote K17 expression, but previous studies in HPV16 transgenic mice revealed a connection between HPV E7’s interaction with pRB and K17 expression (59, 91). The increase in K17 by MmuPV1 E7 raises new questions about the impact of MmuPV1 E7 on pRB and the potential overlap in activities between HPV and MmuPV1 E7. Future studies comparing the MmuPV1 E7-pRB binding mutant and WT MmuPV1 E7 would determine the impact of MmuPV1 E7 on K17 expression.

We have observed that MmuPV1 E7 promotes mTOR signaling in the mouse keratinocyte model system at the transcriptional level and increases the abundance of markers of mTOR signaling, such as phosphorylation of S6 kinase (Fig. 6). Previous work has linked the increase in mTOR signaling to activities of HPV E6/E7 by increasing levels of IQGAP1, which potentiates EGFR signaling and leads to increased activation of PI3K-AKT-mTOR signaling (67). We did not observe an increase in the steady-state levels of phosphorylation sites in AKT and ERK, which are known targets of EGFR signaling (Fig. 6). However, MmuPV1 E7 expression does lead to increased sensitivity of EGFR to EGF stimulation following EGF deprivation (Fig. 7). The ability of MmuPV1 E7 to potentiate EGFR and mTOR signaling is promoted by “high-risk” HPV E6 and E5 oncogenes (66, 67, 72–74). MmuPV1 infection has been found to elevate the protein abundance of IQGAP1 in mice (67). However, this study did not validate this observation that MmuPV1 E7 was responsible for promoting IQGAP1 expression. We do see increased levels of IQGAP1 in our proteomics of MmuPV1 E7-expressing MKs, but we did not confirm this observation in our analysis. Future studies would be needed to connect MmuPV1 E7 to elevated IQGAP1 levels. Importantly, our interactome network analysis did find an enrichment of mTORC1 signaling in the Steiner nodes, which are nodes that parsimoniously bridge between E7 cellular interacting proteins and differentially expressed genes (Fig. 8). Of note, MTOR and RPTOR genes are Steiner nodes that are connected to the MmuPV1 E7 interacting partners MAPKAP1 and RPS6KB1 (S6 kinase). MAPKAP1 is a subunit of mTOR complex 2 that acts as a scaffold protein and assists with substrate specificity of the mTOR catalytic subunit. It promotes metastasis, proliferation, and invasion in some cancers (68, 92, 93). RPS6KB1 is a ribosomal serine/threonine kinase that induces cell growth and proliferation and potentiates survival of some cancer subtypes (94–98). The interaction between MmuPV1 E7 and RPS6KB1 or MAPKAP1 would need to be validated in appropriate cell lines to determine if these are direct interactions. Further studies are needed to determine the impact of MmuPV1 E7 on MAPKAP1, RPS6KB1, and mTOR signaling.

It should be noted that our proteomics data found “Positive Regulation Of Macroautophagy” to be positively enriched (Fig. 3A). This is an interesting observation, as mTOR is an inhibitor of autophagy. This enrichment of autophagy-related proteins is driven by a small subset of proteins that were detected in our proteomic analysis, including GPSM1, SESN2, and SNX18. This is not entirely unexpected, as other studies have shown that HPV also impacts autophagy. Studies on HPV16 E7 have found that E7 inhibits autophagy in head and neck cancer, which increases the sensitivity of these cells to radiation (99, 100). This is in contrast to our own results, where we observe an enrichment of autophagy-related proteins. However, it remains unclear from our analysis if autophagy is activated or inhibited in the presence of MmuPV1 E7. Future studies are needed to address this question.

In conclusion, the data generated in this study have provided new and exciting evidence that MmuPV1 E7 promotes phenotypes in MKs that are associated with “high-risk” HPV infection. While the biochemistry and molecular mechanisms of MmuPV1 E7 mostly mimic observations by cutaneous HPVs, our analysis challenges this hypothesis (53–55). The ability of MmuPV1 to promote infection and disease development, including cancer, at mucosal and cutaneous sites makes our observations not entirely unexpected (45–49). The categorization of MmuPV1 as solely a mimic of cutaneous HPVs may not entirely reflect the complex nature of the MmuPV1 model system. While interacting partners and biochemical activities of MmuPV1 E7 and E6 more reflect their cutaneous counterparts, the increasing evidence showing phenotypes commonly seen in mucosal disease and not cutaneous HPV disease suggests that MmuPV1 should not be categorized as one or the other (54, 56). However, future studies further examining the role of MmuPV1 E7 in MmuPV1-associated mucosal disease are needed to shore up these observations, and comparative studies between the MmuPV1 and HPV model systems are needed to better understand the utility of the MmuPV1 model system as a model for HPV-associated disease, including mucosal disease.

## MATERIALS AND METHODS

### Cells

NIH 3T3 murine fibroblasts were a gift from Dr. Paul Lambert (obtained through ATCC) and grown in Dulbecco’s modified Eagle medium (DMEM) containing 10% bovine calf serum containing Pen/Strep (Gibco). 293FT were a gift from Dr. Paul Lambert (obtained through ATCC) and grown in DMEM containing 10% FBS containing Pen/Strep antibiotic with 200 µg/mL G418. Mouse keratinocytes were isolated from the skin of four different neonate mice on the FVB/N background as previously described to create biological quadruplicate (53, 54, 57). Briefly, neonate mice were euthanized and soaked in betadine for 10 minutes. Following betadine treatment, euthanized pups were soaked in PBS containing 10% antibiotics twice before soaking in 70% ethanol. Skins were isolated and then washed in PBS containing 10% antibiotics. Skin tissue was then soaked in 0.25% trypsin-EDTA overnight at 4°C. The following morning, the epidermis and dermis were separated using sterilized forceps, minced with a single-edge razor blade, and then stirred for 1 hour at 37°C to generate a cell suspension in F-media. The cell suspension was strained using a 0.7 mm membrane (Corning) and cultured in F-media containing 10 mM ROCK inhibitor (Y-27632) in the presence of mitomycin C (MMC)-treated J2 3T3 fibroblasts (57). Early-passage mouse keratinocytes were transduced in keratinocyte serum-free medium (KSFM) (Gibco) with retroviruses encoding MmuPV1 E7 or vector control (pLXSP) (a gift from Dr. Karl Munger) (57). Transduced cells were put under selection 48 hours following transduction with 1 µg/mL of puromycin (Gibco). Following selection, MKs were cultured in F-media containing ROCK inhibitor (Y-27632) with 1 µg/mL of puromycin.

### Plasmids

pLXSP MmuPV1 E7 (mE7) and pLXSP were gifts from Dr. Karl Munger (Tufts University). pCL-10A1 (Fisher Scientific) was used for packaging of pLXSP retroviral vectors and was co-transfected into 293FT cells with pLXSP retroviral vectors (57).

### Retrovirus production

293FT cells were plated into 60 mm tissue culture dishes 24 hours prior to transfection. Plated 293FT cells were changed to DMEM with 10% FBS without antibiotics. 293FT cells were transfected with pLXSP or pLXSP-MmuPV1 E7 and pCL-10A1 vectors using the Lipofectamine 2000 system (Invitrogen) following the manufacturer’s instructions. Twenty-four hours post-transfection, 293FT cells were switched to 3 mL of KSFM, which was used for transductions. Forty-eight hours post-transfection, the supernatant was collected, centrifuged to remove cellular debris, and filtered using a 0.2 µm sterile filter (Thermo Fisher). The cleared supernatant was used for retroviral transductions.

### RNA isolation and RT-PCR

Following selection and establishment of MmuPV1 E7-expressing MKs, cells were collected and lysed in TRIzol Reagent (Invitrogen). RNA was isolated using the Direct-zol RNA MiniPrep Kit (Zymo Research) as we have previously published (57). cDNA was produced using RNA isolated from MKs using the QuantiTect Reverse Transcription Kit (Qiagen) using the manufacturer’s protocol, as we have previously described (57). Briefly, we treated RNA with DNase to eliminate cellular DNA, and DNase-treated RNA was subjected to the RT reaction as described in the manufacturer’s protocol. cDNA generated was used to perform PCR for MmuPV1 E7 and GAPDH or PGK1 (mouse-specific) as internal controls using the primers listed in Table 3. PCRs were run on a 2% agarose gel, which was stained with ethidium bromide, and imaged using the Chemi Doc Imaging System (Bio-Rad).

**TABLE 3:** Primers used in qRT-PCR analysis^*a*^

Primer	Forward	Reverse
MmuPV1 E7	5´-GCGGGCAGACAAAGCTAAGA-3´	5´-GCGACACTGTTCTCCGGTTC-3´
MCM2	5´-CGGAGTATGCGCAAGACTTT-3´	5´-GCCACCAACTGCTTCAGTAT-3´
MCM7	5´-GAGGCCAGCAGATGTGATATT-3´	5´-GGTGTGAAGCCACGAGATATG-3´
CCNE2	5´-ATTTGGCTTTGCTGAATGAAGT-3´	5´-CAGTACTCTTTGGTGGTGTCATA-3´
PCNA	5´-GTTGTCACAAACAAGTAATGTGGAT-3´	5´-CTCAGAAACGTTAGGTGAA-3´
AKT1	5´-GGACTACTTGCACTCCGAGAAG-3´	5´-CATAGTGGCACCGTCCTTGATC-3´
PGK1	5´-GATGCTTTCCGAGCCTCACTGT-3´	5´-ACCAGCCTTCTGTGGCAGATTC-3´
GAPDH	5´-GGAGAGTGTTTCCTCGTCCC-3´	5´-ACTGTGCCGTTGAATTTGCC-3´

^*a*^ Forward and reverse primers are shown for each qRT-PCR that was performed.

RNA isolated from MmuPV1 E7-expressing cells was used for RNA-seq analysis as described below. The remaining RNA was used to generate cDNA for qRT-PCRs for AKT- and E2F-responsive genes, as previously described (57). cDNA was generated from RNA isolated from cells as described above. For qRT-PCR, SYBR Green (Bio-Rad) was used per the manufacturer’s protocol. Primers used for qRT-PCR analysis are described in Table 3.

### Cell counting assays

We utilized our previously published methods for both the long- and short-term growth analyses (57). For the short-term growth analysis, we plated 0.25 × 10^5^ cells into four wells of a six-well plate that contained MMC-treated J2 3T3s. Cells were then counted daily for 4 days. At each time point, feeder cells were removed using 0.05% trypsin-EDTA (Gibco). Following removal of feeders, keratinocytes were washed with Dulbecco’s phosphate-buffered saline (DPBS) and then trypsinized. Cells were resuspended in a total volume of 1 mL and then counted using trypan blue staining to determine live cells on a hemocytometer. Average cell number was plotted. For long-term growth analysis, we plated 1 × 10^5^ cells into a 60 mm dish containing MMC-treated J2 3T3s. Every 3 days, cells were counted. Briefly, plates were treated with 0.05% trypsin-EDTA to remove feeder cells. Following the removal of feeder cells, keratinocytes were washed with DPBS and then trypsinized. Cells were then resuspended in a total volume of 1.5 mL and counted using trypan blue staining to determine live cells on a hemocytometer. Total cell number was determined by multiplying the cell concentration by 1.5. At each time point, the total fold increase in cell number was determined, and the average cumulative fold increase was plotted. Cells were maintained in F-media without ROCK inhibitor for all the counting assays. All assays were performed in biological quadruplicate. For our MK model, a biological replicate is a population of MKs isolated from a single mouse and grown in isolation. Therefore, we generated MmuPV1 E7-expressing MKs from cells isolated from four individual mice, with vector control cells generated from the same population.

### Low-EGF growth assay

Cells were plated at 0.5 × 10^5^ in three wells of a six-well plate containing feeders. After 18 hours, cells were counted to determine the initial number of cells plated at the time of media switch to low-EGF F-media. The other two wells were rinsed twice with 1 mL PBS. One well was treated with F-media, and the other had F-media with reduced FBS (1%) and without epidermal growth factor (low-EGF F-media). After 48 hours cells were counted as described above in 4 day cell counting. Fold increase in cell number was determined following counting, and average fold increase in cell number was plotted.

### EGF-stim

A total of 1 × 10^5^ cells were plated in F-media on three 60 mm dishes and grown to near confluency. Once near confluency, two plates were washed three times with 1 mL PBS. Three milliliters of low-EGF F-media was added to each plate and allowed to grow overnight. Feeder cells were removed from the other plate using 0.05% trypsin-EDTA (Gibco) and washed twice with 1 mL PBS. One hundred fifty microliters of RIPA buffer containing 1:100 Phosphatase cocktail 2 and 3 (Millipore Sigma) and 1:7 cOmplete, Mini, EDTA-free Protease Inhibitor (Millipore Sigma), per the manufacturer’s protocol, was added to each plate, and cells were scraped and collected. The following morning, feeder cells were removed from both plates and rinsed with 1 mL PBS. One plate was harvested with the RIPA mix. The other plate was treated with 3 mL low-EGF F-media spiked with 10 ng/mL EGF. Cells were incubated at 37°C for 5 minutes and immediately placed on ice. Cells were washed twice with 1 mL cold DPBS and lysed with 150 µL of RIPA mix. Lysates were subjected to immunoblot analysis as described below.

### Immunoblotting

Immunoblotting was performed as described previously (57). Briefly, confluent samples were collected and lysed in RIPA buffer containing phosphatase cocktails and protease inhibitors, as described above. Protein concentration was determined within the lysates using Bradford assay (Bio-Rad) per the manufacturer’s protocol. Equivalent amounts of protein were separated in sodium dodecyl sulfate polyacrylamide gel electrophoresis and transferred to nitrocellulose membranes. Membranes were blocked in 5% milk or 5% bovine serum albumin (BSA) (for phosphorylated antibodies) for 1 hour and then incubated overnight with the appropriate primary antibodies diluted in 5% milk or 5% BSA (for phosphorylated antibodies) in 1× PBS and 0.1% Tween 20 (PBS-T). The primary antibodies used are described in Table 4. After being washed, the membranes were incubated with the appropriate horseradish peroxidase-conjugated secondary antibodies (Rockland Immunology) in 5% milk in PBS-T for 1 hour at room temperature and then washed again. Bound antibodies were visualized using enhanced chemiluminescent reagent (Thermo Fisher) according to the manufacturer’s instructions.

**TABLE 4:** Antibodies used in western blot analysis^*a*^

Antibody	Clone	Manufacturer	Dilution
Krt17	EP1623	Abcam	1:1,000
Krt16	8L6R4	Invitrogen	1:1,000
Krt6a	Rb Poly	Protein Tech	1:50,000
Krt6b	Rb Poly	Protein Tech	1:5,000
P-S6	D57.2.2E	Cell Signaling	1:1,000
S6	5G10	Cell Signaling	1:1,000
P-AKT (S473)	D9E	Cell Signaling	1:2,000
Akt	Rb Poly	Cell Signaling	1:1,000
P-ERK1/2 (T202/Y204)	D13.14.4E	Cell Signaling	1:2,000
ERK1/2	137F5	Cell Signaling	1:1,000
Actin	SP124	Abcam	1:2,000
GAPDH	14C10	Cell Signaling	1:1,000

^*a*^ Shown are antibodies used in our immunoblot analysis, with clone, manufacturer, and dilution.

### Proteomic analysis

Once the expression of MmuPV1 E7 was confirmed, frozen cell pellets were subjected to proteomic analysis, specifically quantitative mass spectrometry. Frozen keratinocyte cell pellets were resuspended in lysis buffer (6 M guanidine hydrochloride, 100 mM Tris, pH 8) and probe-sonicated (Misonix) until homogenized. Proteins were then precipitated by adding methanol to the solution to 90% and centrifuging at 10,000 × *g* for 5 minutes. The supernatant was discarded, and the protein pellets were resuspended in digestion buffer [8 M urea, 10 mM tris(2-carboxyethyl) phosphine (TCEP), 40 mM chloroacetic acid (CAA), 100 mM Tris] and sonicated in a bath sonicator for 5 minutes at 10°C with a program of 10 seconds off/20 seconds on (Qsonica). Endoproteinase Lys-C was added in a 100:1 protein:enzyme ratio, and the samples were incubated at room temperature for 4 hours. The samples were then diluted to 1.5 M urea with 100 mM Tris, and trypsin (Promega) was added in a 50:1 protein:enzyme ratio. After overnight incubation at room temperature, the resulting peptides were acidified to pH 2 with trifluoroacetic acid(TFA), desalted with Strata-X Polymeric solid-phase extraction cartridges (Phenomenex), and dried under vacuum.

Each dried peptide sample was resuspended in 0.2% formic acid and loaded onto a 75 µm ID × 360 µm OD capillary column (New Objective) that was packed in-house with 1.7 µm BEH C18 particles (Waters). Chromatographic separations were performed with a Dionex UltiMate 3000 nano HPLC system (Thermo Scientific). The peptides were loaded in 100% A (0.2% formic acid in water) and eluted with increasing % B (0.2% formic acid in 80% acetonitrile [ACN]) over a 90 minute gradient. Mass spectrometric detection was performed with an Orbitrap Eclipse (Thermo Scientific), with MS1 scans taken in the Orbitrap (240,000 resolution, 300–1,350 m/z scan range, 50 ms maximum injection time, and 1 × 10^6^ automatic gain control [AGC] target) and MS2 scans in the ion trap (turbo mode, 0.5 m/z isolation width, 150–1,350 m/z scan range, 14 ms maximum injection time, and AGC target of 3 × 10^4^).

Mass spectrometry raw files were processed with MaxQuant (version 1.5.2.8) and searched against a database of reviewed mouse proteins plus isoforms (downloaded from UniProt on 12 September 2021). Default parameters were used. Cysteine carbamidomethylation and methionine oxidation were set to fixed and variable modifications, respectively. The “proteinGroups.txt” file was processed by omitting reverse sequences, sequences only identified by site, and contaminants. The data were log2-transformed, and proteins were removed if they were not observed in at least 70% of the samples. Remaining missing values were imputed using Perseus (version 1.6.0.7). Statistical comparisons between groups were performed with two-tailed Student’s *t*-tests. Volcano and enrichment plots were made using RStudio (101) with the ggplot2 (102) and ggrepel (103) packages.

### RNA-seq analysis

Following confirmation of expression of MmuPV1 E6 or E7, cells were lysed in TRIzol Reagent (Invitrogen) and total RNA isolated with the Direct-zol RNA MiniPrep Kit (Zymo Research). RNA quality was assessed with a 4200 TapeStation (Agilent) through the UW-Madison Biotechnology Center. Ribo-depletion, library preparation, and RNA-seq on a NovaSeq 6000 was performed by the Oklahoma Medical Research Foundation Clinical Genomics Center (Oklahoma City, OK). A hybrid index was created by the addition of MmuPV1 E7 sequences to GRCm39. Sequencing reads were aligned to the hybrid index using Docker on HTCondor through the UW-Madison Center for High Throughput Computing using STAR 2.7.6a (104, 105). Read summarization was performed with featureCounts v.1.6.3 and Ensembl annotation release 105. Differential expression analysis was performed with DESeq2 v.1.34.0. Gene set enrichment analysis was performed with GSEA v.4.3.2 and the Hallmark Gene Set Collection from MSigDB (70, 106, 107). Volcano and enrichment plots were made using RStudio (101) with the ggplot2 (102) and ggrepel (103) packages.

### STRING analysis

From the RNA-seq and quantitative mass spectrometry analysis, the list of genes/proteins that met the requirement of log2(FC) > 1 or log2(FC) < −1 and *P*_adj_ <0.05 was generated. Each list of genes was then separated into upregulated [log2(FC) > 1] or downregulated [log2(FC) < −1] and uploaded to the STRING database (string-db.org). Following the uploading of gene sets, STRING interaction networks were generated for each gene/protein list with parameters set to highest confidence, no text mining, and hiding unconnected nodes. Nodes were pseudo-colored red for upregulated and blue for downregulated. Images of networks were downloaded for visualization.

### Interactome network analysis

DESeq2 differentially expressed genes with *P*_adj_ < 0.05 were connected to MmuPV1 E7 interactors through the STRING protein-protein interaction network using the R package PCSF (108). The PCSF algorithm uses message passing to identify high-confidence subnetworks that contain the minimal set of edges connecting user-defined terminal nodes (i.e., the experimentally observed genes and proteins) through bridging nodes (also called Steiner nodes) that are not experimentally observed. PCSF was run using default parameters. Functional enrichment was run on the terminal nodes and Steiner nodes using g:Profiler (109).

### Statistics

All statistical tests were done using MStat software (https://oncology.wisc.edu/mstat/), GraphPad Prism (growth curves), or Microsoft Excel.

## Data Availability

All data are available in public databases. MS raw files were deposited to the MassIVE database with the identifier MSV000089441. The RNA-seq data reported in this paper are available in the BioProject database with the accession numbers PRJNA1007707 and PRJNA1322560.

## SUPPLEMENTAL MATERIAL

Figure S1 jvi.01097-25-s0001.pdf MmuPV1 E7 promotes EGFR signaling without altering AKT expression.

Supplemental tables jvi.01097-25-s0002.xlsx Tables S1 to S3.

ASM does not own the copyrights to Supplemental Material that may be linked to, or accessed through, an article. The authors have granted ASM a non-exclusive, world-wide license to publish the Supplemental Material files. Please contact the corresponding author directly for reuse.
