# Supplementary material for: MmuPV1 E7 promotes phenotypes associated with “high-risk” HPV infection in mouse keratinocytes
Source: J Virol. 2025 Oct 30;99(11):e01097-25. doi: 10.1128/jvi.01097-25 (PMC12645909; doi:10.1128/jvi.01097-25)
Supplement: Figure S1 — MmuPV1 E7 promotes EGFR signaling without altering AKT expression. [file jvi.01097-25-s0001.pdf]

Supplemental Figure 1

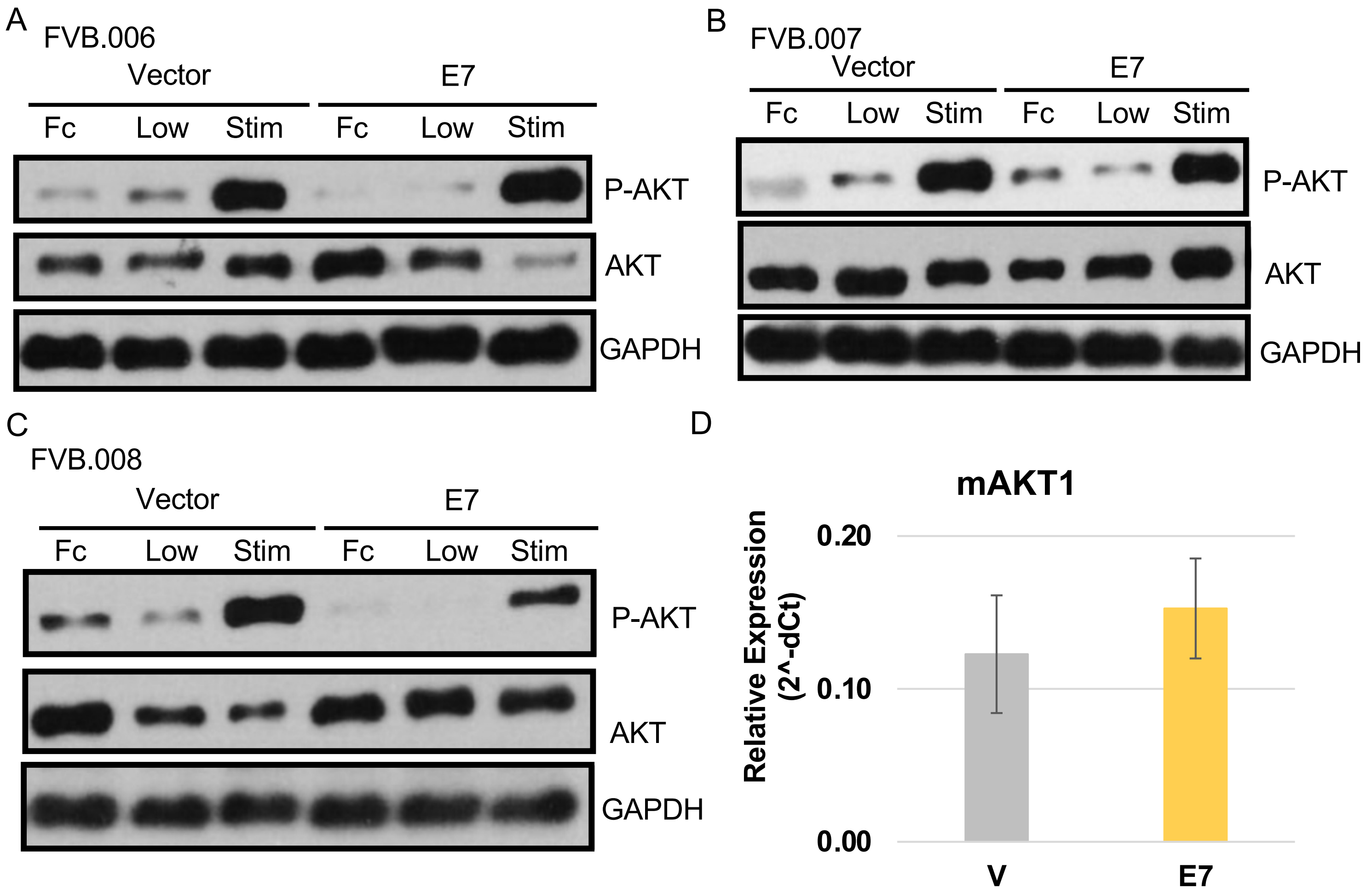

Supplemental Figure 1: MmuPV1 E7 promotes EGFR signaling without altering AKT expression. A-C) Western blot analysis of MKs treated with EGF following depravation. Each set of images is a biological replicate from the analysis. **D)** qRT-PCR analysis for AKT in our mE7 versus vector control MKs. Analysis was performed in biological quadruplicate (n=4).
